# Social and Poor-Law Problems

**Published:** 1908-01-04

**Authors:** 


### SOCIAL AND POOR-LAW PROBLEMS.

### IN CASES OF EMERGENCY, NURSING AND OBSTETRIC.

At a conference recently held between the Birmingham Health Committee and the Guardians of the component parishes some questions were raised in connection with the Midwives Act. In certain circumstances it is the duty of a midwife to call in the assistance of a registered medical practitioner; but it was pointed out that sometimes the midwife finds difficulty in getting a doctor to come at her request. This seems strange, seeing that a doctor must know that when a midwife sends for him the life of a mother and perhaps that of her child are in danger. It was suggested that the parish doctors should have instructions to honour the request of a midwife for help as if it were that of a relieving officer. Whether or not the parish doctor be so instructed, it is necessary that a midwife should be sure of getting the help she needs in a difficult case, even though the assistance given should be paid for out of public funds.

Lately the Clerk to the Tetbury Union wrote to the Local Government Board asking if a medical officer attending a pauper midwifery case would be permitted to call in a trained nurse from a distance to nurse the case on the ground that skilled nursing was necessary and that there was, in his opinion, no person in the neighbourhood capable of properly undertaking the work; and if the medical officer gave such a recommendation, was the relieving officer bound to comply with it and the Guardians to pay the nurse. The reply of the Board was that, though the medical man's recommendation had not the force of an order, which should be given by the relieving officer or the Guardians, "in the absence of very special reasons, they would incur a grave responsibility in declining to provide skilled nursing at the medical officer's request."

